# Kindness Should Still Underline Science During the Pandemic

**DOI:** 10.1002/bes2.1757

**Published:** 2020-07-13

**Authors:** Francisco E. Fontúrbel, Jeferson Vizentin‐Bugoni

**Affiliations:** ^1^ Instituto de Biología Facultad de Ciencias Pontificia Universidad Católica de Valparaíso Avenida Universidad 330 Valparaíso 2373223 Chile; ^2^ Department of Natural Resources and Environmental Sciences University of Illinois at Urbana‐Champaign Turner Hall Urbana Illinois 61820 USA

The COVID‐19 pandemic has changed most people's lives in many ways, and scientists are no exception (Inouye et al. 2020, Staniscuaski et al. 2020). Scientists have to deal with a new reality that includes teaching and leading their research groups from home, which may be challenging to balance with housekeeping and parenting (Staniscuaski et al. 2020). Such an unprecedented workload, combined with the uncertainty of the short‐term future, particularly funding and the progress of ongoing research projects, is wearing scientists' nerves thin. A less evident side effect of this new reality is the unspoken increase of grumpiness in the publishing process. While exhausted authors are less effective with the meticulous wordsmithing of their manuscripts, exhausted reviewers may be less tolerant than usual with minor issues, and exhausted editors may be more prone to reject manuscripts that could be easily fixed after a revision, or have to work even more to guarantee fair reviews (i.e., editing or re‐doing the review themselves or finding new reviewers). This may delay the publication process, which most journals have acknowledged on their websites. This may also affect disproportionally certain groups of scientists, especially early career researchers who are not native English speakers, based in institutions that are facing cuts on the already meager budget once used to cover scientific editing services. While we do not advocate relaxing the rigor of the peer review process, we point out that this less evident consequence of the pandemic is likely to impact scientific production between 2020 and 2021, making particularly vulnerable researchers from less‐developed countries, and those overloaded with child care and eldercare. To prevent aggravating inequalities in the global scientific landscape, we reiterate the need for editors and reviewers to be as empathic with authors as in prepandemic times.
